# Beyond Incidentalomas: Hemodynamic Assessment and Minimally Invasive Management of Pericardial Cysts

**DOI:** 10.1016/j.jaccas.2026.109760

**Published:** 2026-08-19

**Authors:** Antonio Carlos Escorel de Almeida Neto, Luiz Rafael Pereira Cavalcanti

**Affiliations:** Department of Cardiovascular Surgery, IMIP, Recife, Pernambuco, Brazil

**Keywords:** cardiac magnetic resonance, chest pain, imaging, robotic surgery

**Figure:**
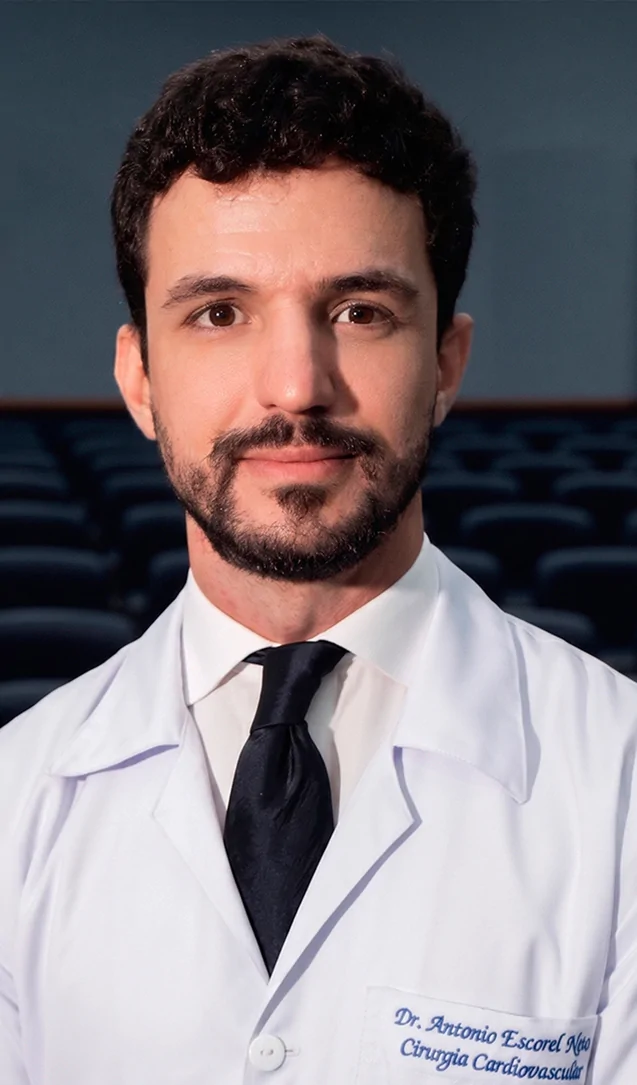

Pericardial cysts are uncommon, benign anomalies of the mediastinum, typically arising from the incomplete coalescence of fetal lacunae during pericardial development. Most cysts are discovered incidentally and remain asymptomatic; however, a subset enlarges and causes mechanical compression.^1^ Symptoms typically include chest pain, dyspnea, and arrhythmias. In this context, the decision to transition from conservative observation to surgical intervention requires objective evidence of anatomical impingement or hemodynamic compromise.

In this issue of *JACC Case Reports*, German et al^2^ present the case of a 47-year-old man with progressive exertional dyspnea, cough, and chest tightness. Diagnostic imaging identified a large (7.9 × 4.0 × 6.7 cm) pericardial cyst compressing the right ventricular apex. The authors managed the patient with a left-sided video-assisted thoracoscopic surgery (VATS) resection, resulting in complete symptom resolution. This case illustrates the clinical pathway for managing symptomatic extrinsic cardiac compression.

A notable element of this case is the documentation of a reduced pulmonary artery pulsatility index (PAPi) of 2.7 during right heart catheterization. PAPi is a recognized hemodynamic marker of right ventricular dysfunction, initially validated in the setting of acute myocardial infarction and advanced heart failure.^3^ Its reduction in this patient, who lacked primary cardiopulmonary disease, indicates an extrinsic mechanical restriction of right ventricular filling and stroke volume. When structural compression from a mediastinal mass restricts the right ventricle, it can mimic primary right heart failure. Objective metrics like PAPi can corroborate a patient's exertional symptoms and provide a physiological justification for surgical intervention.

Accurate preoperative planning relies on advanced multimodality imaging. Although transthoracic echocardiography remains the initial diagnostic modality, cardiovascular magnetic resonance (CMR) is the definitive standard for evaluating cardiac masses.^4^ CMR provides high spatial resolution and tissue characterization, differentiating fluid-filled, avascular cysts from solid neoplasms or teratomas.^4^ As in the literature on cardiac masses, pericardial cysts typically exhibit low signal intensity on T1-weighted images, high signal intensity on T2-weighted images, and an absence of late gadolinium enhancement.^4^ The protocol utilized by German et al^2^ aligns with these standards, confirming the benign nature of the lesion and defining its physical relationship with the pericardium and myocardium before intervention.

The surgical management of pericardial cysts has evolved significantly. Historically, percutaneous aspiration was considered an alternative to open surgery; however, it is associated with recurrence rates approaching 30%.^1^ Consequently, surgical resection is the definitive treatment. VATS has largely replaced open thoracotomy as the standard of care for mediastinal mass resection, offering reduced postoperative complications, less pain, and lower overall morbidity.^5^ The left-sided VATS approach selected by the authors demonstrates the necessity of tailoring the surgical access to the patient's specific anatomy. Guided by preoperative CMR, this approach avoided unnecessary manipulation over the anterior cardiac surface.

The report by German et al^2^ reinforces a structured approach to symptomatic pericardial cysts. Integrating multimodality imaging for tissue characterization, acknowledging the hemodynamic consequences of right ventricular compression, and using targeted minimally invasive surgical techniques ensures optimal and definitive clinical outcomes.

## Funding Support and Author Disclosures

The authors have reported that they have no relationships relevant to the contents of this paper to disclose.
